# Haemodynamic monitoring as an opportunity for tailoring diuretics and guideline‐directed medical therapy in heart failure

**DOI:** 10.1002/ctm2.1372

**Published:** 2023-09-15

**Authors:** Jishnu Malgie, Jasper J. Brugts, Rudolf A. de Boer

**Affiliations:** ^1^ Department of Cardiology Erasmus MC University Medical Centre Rotterdam The Netherlands

1

Heart failure (HF) is prevalent and associated with a significantly impaired quality of life, frequent hospitalisations and a high mortality rate. Fortunately, advances in pharmacological HF treatment, particularly for heart failure with reduced ejection fraction (HFrEF), have significantly improved patient care. Guideline‐directed medical therapy (GDMT) forms the cornerstone of HFrEF therapy and currently consists of four drug classes: renin‐angiotensin system inhibitors (RASi), beta‐blockers (BB), mineralocorticoid receptor antagonists (MRA), and sodium‐glucose cotransporter‐2 inhibitors (SGLT2i). However, the basis remains that GDMT is tailored on top of optimised doses of diuretics and an euvolemic fluid state.

HF is characterised by periods of worsening with fluid retention. As congestion worsens, hemodynamic pressures rise, followed by typical HF signs and symptoms such as orthopnea, dyspnea, and increased venous jugular distention. Unfortunately, most signs and symptoms are aspecific and manifest late in the deterioration process. This often occurs between outpatient clinic visits ‐ due to suboptimal monitoring and management at home ‐ and can result in hospitalisations. Remote hemodynamic monitoring modalities, such as CardioMEMS, can detect congestion before typical clinical symptoms arise. The small sensor is implanted in the pulmonary artery (PA) and can provide remote measurements of PA pressure (PAP) as a surrogate marker of filling pressures and fluid state. PA pressure is a clinically intuitive and interpretable parameter to monitor remotely. This approach allows for pre‐emptive interventions in case of high or low PAP, mainly by adjusting loop diuretics, to prevent further congestion and hospitalisations.

The first two landmark CardioMEMS trials, CHAMPION and GUIDE‐HF, analysed the efficacy of CardioMEMS in North America.^1^, ^2^ While CHAMPION included NYHA III patients with one previous HF hospitalisation, GUIDE‐HF encompassed a broader population of NYHA II‐IV patients with either a previous HF hospitalisation or only an elevated NT‐proBNP. The MONITOR‐HF trial tested the use of CardioMEMS on quality of life and HF hospitalisations in a different healthcare system in Europe.^3^ The trial was conducted in the Netherlands and included NYHA class III patients across the ejection fraction spectrum, with a history of HF hospitalisation in the past 12 months. The trial had an open‐label design and randomised 348 patients to either CardioMEMS or usual care. Patients had a median age of 69 years and a median ejection fraction of 30%. GDMT use at baseline was relatively high compared to CHAMPION and GUIDE‐HF, with adherence rates exceeding 80% for RASi, BB, and MRA. SGLT2i prescription was comparatively low as the trial started well before these drugs were recommended in HF guidelines, but uptake in both groups during the trial was remarkable with roughly 30% on SGLT2i at 12 months.

The primary outcome of MONITOR‐HF was quality‐of‐life as measured by the Kansas City Cardiomyopathy Questionnaire (KCCQ) overall summary score, which showed a significant improvement in favour of CardioMEMS and no change in patients randomised to standard care. The CardioMEMS group experienced significantly fewer hospitalisations compared to the control group. The benefits found in the CardioMEMS group extended to multiple levels such as a substantial decline in mean PAP from 33 to 25mmHg at 12 months and substantial reductions in levels of NT‐proBNP as well. Changes in especially loop diuretics occurred more often in the CardioMEMS group compared to the control group, both in terms of intensifications and downgrades. Also, the cumulative number of changes in GDMT was higher in patients with CardioMEMS. Still, GDMT changes were at much lower absolute numbers (with 4 components of GDMT drugs combined) as compared to diuretics. Additionally, there was no significant difference in GDMT prescription rates at 12 months follow‐up between groups.

A meta‐analysis of the three CardioMems trials—CHAMPION, GUIDE‐HF, and MONITOR‐HF—reported the use of CardioMEMS to result in a significant reduction in HF hospitalisations, with a hazard ratio of 0.70 (95% confidence interval 0.58–0.86).^4^ This pooled effect shows the potential for CardioMEMS across multiple countries with significantly different healthcare systems and against evolving background therapy. However, because the studies were not powered for mortality, mortality benefits are yet to be confirmed.

As emphasised in the discussion of MONITOR‐HF, remote haemodynamic monitoring itself does not treat the patient. Instead, the daily PAP measurements allow for timely, individually tailored, and fine‐tuned medical interventions. Although in the trials the effect of CardioMEMS was primarily driven by changes in loop diuretic dose, the utility of CardioMEMS could extend beyond this with a better decongestive status of the patient. One such intriguing utility could be offering a means to better guide other recommended drugs, an area where improved tools are urgently needed. Multiple large‐scale registries from multiple continents show lacking prescription rates to proven life‐saving drug therapy, with prescribed doses generally well below those recommended by the HF guidelines.^5^

Particularly for the more vulnerable NYHA III worsening‐HF population included in the MONITOR‐HF trial, optimising GDMT prescription and dose are essential when the better fluid monitoring has provided a chronically better decongestive status. The “risk‐treatment paradox” is notably pronounced in this subset of patients, arising from a wide range of factors attributed to the patient, healthcare system, and healthcare provider.^5^ For instance, assumed intolerance or a disproportionate fear of side effects can limit GDMT implementation in frail patients who are most in need of effective medical therapy. Especially for BB, studies have demonstrated improved outcomes with up‐titration. For example, the MOCHA trial showed a dose‐response relationship between carvedilol dose and left ventricular ejection fraction improvement, as well as for hospitalisation and mortality rates.^6^ A common reason for not initiating or not up‐titrating BB is the fear of worsening HF or even cardiogenic shock, presumably caused by the drug's negative inotropic effects. Low PAP values signaling the absence of left‐sided decompensation could potentially take away this fear, reducing the barrier for BB up‐titration, for example. Conversely, increased PAP might provide an opportunity to up‐titrate vasodilators and/or angiotensin receptor‐neprilysin inhibitors or SGLT2i,^7^, ^8^ the vast majority of all drug changes in the trial were diuretics. From that perspective, having a tool that provides information on the fluid status of a patient at home (previously a black box) allows for tailored decongestive therapy and is one of the most relevant achievements of this system. Therefore, this cannot emphasize the importance of diuretics as first and main cornerstone therapy even more.

In conclusion, with the addition of the MONITOR‐HF trial the aggregate data supports haemodynamic monitoring for the management of HF. This is on top of real‐world studies such as COAST and MEMS‐HF, which demonstrate even more proncounced effects on HF hospitalisations.^9^, ^10^ As health care professionals familiarise themselves with additional (haemodynamic) monitoring tools between visits, it could substantially aid in optimising HF care, advancing personalised medical therapy to maximise patient outcomes.

## CONFLICT OF INTEREST STATEMENT

Jishnu Malgie: Nothing to declare. Jasper J. Brugts: Independent research grant from Abbott for ISS to institute and has had speaker engagement or advisory boards in the past 5 years with Astra Zeneca, Abbott, Boehringer Ingelheim, Bayer, Daiichi Sankyo, Novartis and Vifor. Rudolf A. de Boer: Received research grants and/or fees from AstraZeneca, Abbott, Boehringer Ingelheim, Cardior Pharmaceuticals GmbH, Ionis Pharmaceuticals, Inc., Novo Nordisk and Roche; and has had speaker engagements with Abbott, AstraZeneca, Bayer, Bristol Myers Squibb, Novartis and Roche.
